# Tricuspid regurgitation in the context of severe left-sided valvular disease: Patients characteristics and outcome

**DOI:** 10.1016/j.heliyon.2024.e34874

**Published:** 2024-07-19

**Authors:** Davide Margonato, Giacomo Ingallina, Martina Belli, Annamaria Tavernese, Gianluca Ricchetti, Francesco Ancona, Stefano Stella, Federico Biondi, Giorgio Fiore, Michele De Bonis, Francesco Maisano, Yan Topilsky, Eustachio Agricola

**Affiliations:** a Cardiovascular Imaging Unit, Cardiothoracic Department, IRCCS San Raffaele Scientific Institute, Milan, Italy; b Cardiac Surgery Unit, Cardiothoracic Department, IRCCS San Raffaele Scientific Institute, Milan, Italy; c Vita-Salute University San Raffaele, Milan, Italy; d Department of Cardiology, Tel Aviv Medical Center, Tel Aviv, Israel

**Keywords:** Tricuspid regurgitation, Left-sided valvular heart disease, Long-term outcome

## Abstract

**Background:**

We aimed to assess the characteristics, management and long-term prognosis of a cohort of patients with multiple valvular disease, focusing on the context of severe mitral or aortic disease with concomitant significant tricuspid regurgitation (TR).

**Methods:**

After using a propensity score matching for age, 975 patients with ≥ moderate TR, diagnosed at our centers from 2012 to 2020, were included and divided in four groups, including isolated TR patients as reference group. Primary endpoint was all-cause death (ACD), secondary endpoint was the composite of heart failure (HF) hospitalization + any valvular intervention.

**Results:**

Patients with isolated TR (356, 37 %) had more history of atrial fibrillation and were more often asymptomatic and with preserved left-ventricular ejection fraction (LVEF). Patients with severe mitral regurgitation (MR) + TR (466, 48 %) showed higher rates of concomitant coronary artery disease, advanced functional class symptoms and larger left atrial volumes. Severe aortic stenosis (AS) patients (131, 13 %) were older, with more comorbidities and lower LVEF. Patients with severe aortic regurgitation and TR (22, 2 %) were younger, with larger LV dimensions and higher pulmonary arterial pressures.

After a median follow-up of 2.8 years, both endpoints were univariably more frequent in patients with severe AS + TR (all p < 0.001), but after comprehensive adjustment difference in the primary endpoint became insignificant, underscoring the serious outcomes of all significant TR groups significantly. Overall, in 44 (5 %) patients tricuspid intervention was performed, with no differences between groups in term of frequency of concomitant or staged tricuspid valve surgical treatment.

**Conclusions:**

In the context of severe left-sided VD, concomitant significant TR is common, and each subtype presents with different clinical and echocardiographic features: patients with severe AS and TR have considerable worse prognosis, although comprehensive adjustment reflected the poor outcomes affecting all types of patients with significant TR. In this scenario, TR was profoundly undertreated.

## 1 Introduction

Tricuspid regurgitation (TR) is a highly prevalent valvular disease (VD) that, when of at least moderate entity, worsens mid and long-term survival regardless of comorbidities [[1], [2], [3], [4], [5]]. Functional changes in the tricuspid valve (TV) apparatus in the setting of pulmonary hypertension (PH) and right ventricle (RV) remodeling due to left-heart disease frequently lead to significant TR: therefore, concomitant left-sided VD often has a crucial role in TR development and progression [[5], [6], [7]]. In the context of multiple VD (MVD), a Swedish hospital register-based study collecting data from the entire Swedish population between 2003 and 2010 [8], showed that out of 2883 TR patients, the percentage of coexisting left-sided VD was 5.4 % for aortic regurgitation (AR), 7.1 % for aortic stenosis (AS) and 28.8 % for mitral regurgitation (MR). In the Valvular Heart Disease Survey II study [9], out of 5087 patients with severe native VD, 15 % presented severe TR and at least one associated severe left-sided VD, and very recent insights from the same registry showed that an increase in TR grade was associated with a more severe clinical presentation and a poorer 6-month survival [10]. Despite the validity of this epidemiologic data, a comprehensive clinical and echocardiographic characterization of patients with severe left-sided VD and concomitant TR is missing, as concerns mostly persist due to heterogeneity of the MVD context.

In the last years, a reappraisal on the therapeutical management of TR has emerged [5,6], particularly due to novel transcatheter strategies, which represent a therapeutical option of particular interest for the typical high-surgical risk cohort affected by TR and MVD. Although current guidelines [11,12] present specific recommendations for intervention in patients with mild, moderate and severe TR undergoing left-sided surgery, both documents highlight the lack of data focusing on the prognosis and on the most appropriate therapeutical management of concomitant multiple VD; as a consequence of this conundrum, patients with severe MVD undergo far fewer valvular intervention as compared to single VD patients, despite comparable age [9].

To address these gaps of knowledge, here we report the clinical profile, the outcome and the therapeutical management of a large cohort of patients with MVD, focusing on the specific context severe mitral or aortic disease and concomitant significant TR.

## 2 Methods

This is a retrospective multicenter study. Patients considered eligible were those with a diagnosis of at least moderate TR identified on echocardiography between January 2012 and June 2020 at San Raffaele Hospital (Milan, Italy) and at Tel-Aviv Medical Center (Tel-Aviv, Israel), along with a comprehensive clinical and echocardiographic evaluation. Exclusion criteria were the absence of clinical follow-up information, a history of previous TV intervention (either surgical or percutaneous), the presence of concomitant TV stenosis and of congenital heart disease. The study protocol conforms to the ethical guidelines of the 1975 Declaration of Helsinki and was approved by our institutional review board (LOCOMOTRI study protocol). This research was done without patient involvement.

A complete clinical evaluation for symptoms, functional New York Heart Association (NYHA) class, major comorbidities (summed as Charlson Comorbidity Index score [CCI]) and data regarding baseline haemoglobin and glomerular filtration rate were collected. A history of chronic kidney disease was defined as an estimated glomerular filtration rate <60 mL/min/1.73 m^2^ for at least 3 months. Anemia was defined as haemoglobin levels <12.0 g/dL in women and <13.0 g/dL in men according to World Health Organization definition [13]. Information regarding any valvular intervention during follow-up was also assessed.

Complete transthoracic echocardiograms were performed at rest with commercially available ultrasound system (Vivid E9 e Vivid E95, General Electric Healthcare, Milwaukee, WI, USA and Epic 7 and IE 33, Philips, Amsterdam, Netherlands). Data was collected from the first available echocardiographic exam during the designed period of investigation and was extracted without alteration or reinterpretation for the study.

VD severity was assessed using a multiparametric approach as recommended by both European and American guidelines [[14], [15], [16]]. Only patients with hemodynamically relevant TR, namely ≥ moderate, were included in the study. The study population was then divided into four groups according to the type of left-sided VD accompanying TR:

- Isolated ≥ moderate TR as reference group. Isolated TR was defined in the absence of any concomitant VD > mild.
- Severe MR+ ≥moderate TR
- Severe aortic stenosis (AS)+ ≥moderate TR
- Severe aortic regurgitation (AR)+ ≥moderate TR

Morphological and functional analysis of heart chambers was assessed according to the EACVI/American Society of Echocardiography consensus document of 2015 [17]; systolic pulmonary artery pressure was calculated using continuous-wave Doppler TR velocity and right atrial (RA) pressure estimated using inferior vena cava imaging.

The primary end point was all-cause mortality, censoring patients at the time of valvular intervention. The secondary end point was the composite of first occurrence of HF hospitalization plus the need for any valvular intervention (either surgical or percutaneous). Follow-up and survival data were extracted from the electronic health records or via phone interview.

Categorical variables were expressed as count (percentage) and compared with the χ2 or Fisher exact test. Continuous variables were expressed as mean (standard deviation) or median (interquartile range, IQR); Student's T and Anova test, or Mann Whitney U and Kruskal Wallis test, were used as appropriate. The predictors of events were identified by performing univariate Cox proportional hazards analysis. The odds ratio (OR) and 95 % confidence interval (CI) were defined. To confirm the independent predictive value, variables with *p <* 0.05 were tested in a multivariate model, with a maximum of 1 covariate per 10 events. When there was a significant correlation between variables, only one of them was included to avoid multicollinearity. Survival and event-free survival were estimated by the Kaplan–Meier method and compared by log-rank test. Analysis was performed by censoring follow-up at time of last follow-up or at the time of event occurred. A two-tailed *p* < 0.05 was considered statistically significant.

Propensity score matching for age was used to create matched cohorts in the different groups of valvopathies. The approach selected was “Exact Matching” and an absolute standardized differences less than <10 % was adopted to indicate no meaningful imbalance. Because of the challenging in exact matching of a continuous variable such as age, we allowed a 10 % age difference: with this approach, a continuous variable is matched between groups without losing an excessive number of patients from each group. Statistical analyses were performed using SPSS 24.0 (SPSS, Inc., Chicago, IL, USA) and R version 3.6.1 software (R Development CoreTeam, Vienna, Austria) with “survminer,” and “matchIT” packages.

## 3 Results

**Fig. 1:**
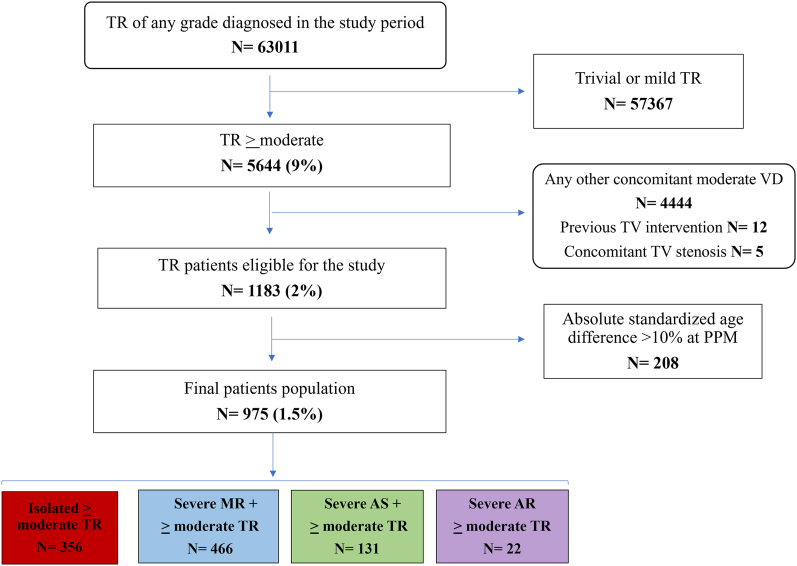
Study population flow chart.

Out of 5644 patients with ≥moderate TR evaluated in the study period, after application of exclusion criteria, 1183 patients were eligible for the study (Fig. 1). Following propensity score matching to reduce significant age imbalance, 975 patients represented our final cohort.

Supplementary Table 1 reports the baseline characteristics of the population before propensity score matching, while supplementary Table 2 describes propensity score matching computation leading to the final enrollable cohort.

The final population included 356 patients with isolated TR (37 %), 466 patients with severe MR+ ≥moderate TR (48 %), 131 with severe AS+ ≥moderate TR (13 %) and 22 with severe aortic regurgitation (AR)+ >moderate TR (2 %).

Overall, mean age was 77 ± 9 years, 52 % female. Twenty-seven percent had a history of coronary artery disease (CAD), 45 % of atrial fibrillation (AF) and 63 % of CKD; mean CCI was 4.3 ± 1.7 and 33 % were in NYHA class III/IV. As for echocardiographic parameters, mean left-ventricular ejection fraction (LVEF) was 51 ± 12 %, mean stroke volume indexed (SVi) 34 ± 11 ml/m^2^ and mean pulmonary artery systolic pressure (PASP) 51 ± 16 mmHg. Baseline clinical and echocardiographic characteristics of the overall population are presented in Table 1.

**Table 1:** Baseline characteristics of the overall population and of patients according to valvular heart disease.

	Overall population (n = 975)	Isolated TR (n = 356, 37 %)	TR + severe MR (n = 466, 48 %)	TR + severe AS (n = 131, 13 %)	TR + severe AR (n = 22, 2 %)	Overall p-value
**Age (years)**	77 ± 9	78 ± 8	76 ± 9	81 ± 9	73 ± 9	**<0.001**
**Female gender, n (%)**	513 (52 %)	235 (66 %)	190 (40 %)	80 (61 %)	8 (36 %)	**<0.001**
**Weight (kg)**	70 ± 14	70 ± 15	70 ± 13	68,7 ± 12	76 ± 30	0.131
**BMI (kg/m**^**2**^**)**	25.6 ± 4.8	26.3 ± 5.6	25.2 ± 4.1	25.4 ± 4.3	25.7 ± 7.4	0.787
**BSA (m**^**2**^**)**	1.7 ± 0.2	1.7 ± 0.2	1.7 ± 0.2	1.7 ± 0.1	1.8 ± 0.3	0.160
**Hypertension**	651 (67 %)	238 (67 %)	307 (66 %)	96 (73 %)	10 (45 %)	0.093
**Diabetes**	239 (24 %)	86 (24 %)	123 (26 %)	32 (24 %)	5 (22 %)	0.541
**Dyslipidemia**	432 (44 %)	157 (44 %)	215 (46 %)	52 (39 %)	8 (40 %)	0.608
**Smoking history (former or current)**	114 (11 %)	43 (12 %)	60 (13 %)	5 (4 %)	6 (29 %)	**0.006**
**History of CAD**	261 (27 %)	73 (20 %)	158 (34 %)	26 (20 %)	4 (18 %)	**<0.001**
**Previous CABG**	33 (3 %)	15 (4 %)	13 (3 %)	4 (3 %)	1 (4 %)	0.608
**Cardiomyopathy**	57 (6 %)	16 (4 %)	36 (7 %)	4 (3 %)	1 (4 %)	0.063
**CIED**	125 (13 %)	46 (13 %)	65 (14 %)	9 (7 %)	5 (22 %)	0.108
**Atrial fibrillation**	441 (45 %)	216 (60 %)	173 (37 %)	47 (36 %)	5 (23 %)	**<0.001**
**COPD**	74 (7 %)	35 (9 %)	30 (6 %)	8 (6 %)	1 (5 %)	0.286
**History of cancer (past or active)**	181 (18 %)	66 (18 %)	87 (18 %)	25 (19 %)	3 (13 %)	0.875
**CKD**	620 (63 %)	222 (62 %)	304 (65 %)	86 (65 %)	8 (36 %)	**0.026**
**Charlson Comorbidity Index**	4.3 ± 1.7	4.4 ± 1.7	4.2 ± 1.7	4.7 ± 1.6	3.6 ± 1.5	**0.002**
**NYHA class**						**<0.001**
**I**	257 (26 %)	170 (48 %)	53 (12 %)	24 (18 %)	10 (45 %)	
**II**	399 (41 %)	114 (32 %)	220 (47 %)	57 (44 %)	8 (37 %)	
**III/IV**	319 (33 %)	72 (20 %)	193 (41 %)	50 (38 %)	4 (18 %)	
**Hb (g/dl)**	10.9 ± 2.1	11.4 ± 2.1	10.5 ± 2.1	10.4 ± 1.9	11.2 ± 2.5	**<0.001**
**Anemia**	711 (73 %)	221 (62 %)	378 (81 %)	102 (78 %)	10 (45 %)	**<0.001**
**eGFR (CKD-EPI, ml/min/mq)**	48 ± 24	51 ± 27	46 ± 26	46 ± 20	57 ± 24	**0.015**
**LVEDD (mm)**	53 ± 6	45 ± 6	57 ± 7	51 ± 6	56 ± 6	**<0.001**
**LVEF (%)**	51 ± 13	54 ± 10	50 ± 15	48 ± 13	48 ± 12	**<0.001**
**≤40 %**	224 (23 %)	47 (13 %)	126 (27 %)	45 (34 %)	6 (27 %)	
**40–49 %**	97 (10 %)	21 (6 %)	60 (13 %)	13 (10 %)	3 (13 %)	
**≥50 %**	654 (67 %)	288 (81 %)	280 (60 %)	73 (56 %)	13 (60 %)	
Stroke Volume Indexed (ml/m2)*	34 ± 11	30 ± 9	34 ± 11	36 ± 11	52 ± 19	**<0.001**
**LAESV indexed (ml/m2)**	58 ± 14	53 ± 14	64 ± 15	58 ± 14	59 ± 14	**<0.001**
**PASP (mmHg)**	51 ± 16	46 ± 13	54 ± 16	55 ± 16,1	58 ± 17	**<0.001**
**35–50 mmHg**	383 (39 %)	174 (49 %)	158 (34 %)	44 (34 %)	7 (31 %)	
**> 50 mmHg**	456 (47 %)	113 (32 %)	256 (55 %)	73 (56 %)	14 (63 %)	
**RVED basal diameter (mm)**	42 ± 8	41 ± 8	44 ± 9	38 ± 8	43 ± 7	0.05
**TAPSE (mm)**	18 ± 5	17 ± 4	19 ± 5	18 ± 4	18 ± 4	0.408
**Severe TR**	471 (48 %)	62 (17 %)	381 (81 %)	26 (20 %)	2 (9 %)	**<0.001**
**RAES area (cm2)**	25 ± 8	29 ± 8	24 ± 7	22 ± 7	23 ± 4	**<0.001**
**RAP (mmhg)**	11 ± 6	13 ± 6	11 ± 5	10 ± 5	11 ± 5	**<0.001**

Values are mean ± SD or n (%), unless otherwise specified. Abbreviations: BMI, Body Mass Index, is the weight in kilograms divided by the square of the height in meters; BSA, Body Surface Area; CAD, Coronary Artery Disease; CABG, Coronary Artery Bypass Graft; CIED, Cardiac Implantable Electronic Device; COPD, Chronic Obstructive Pulmonary Disease; CKD, Chronic Kidney Disease, defined as eGFR <60 mL/min; eGFR, estimated Glomerular Filtration Rate; EPI, Epidemiology Collaboration; LVEDD, Left Ventricular End-Diastolic Diameter; LVEF, Left Ventricular Ejection Fraction; * = 552 patients; LAESV, Left Atrium End-Systolic Volume; PASP, Pulmonary Artery Systolic Pressure; RVED, Right Ventricle End-Diastolic; TAPSE, Tricuspid Annular Plane Systolic Excursion; TR, Tricuspid Regurgitation; RAES, Right Atrium End-Systolic; RAP, Right Atrium Pressure.

Severe TR was present in 473 (48 %) patients, and was significantly more common in severe MR patients compared to all the other groups (see Supplementary Table 3). Concomitant severe TR and RV dysfunction was present in 91 patients (9 %), and 112 (11 %) had concomitant severe TR and CAD. Factors associated with severe TR in all patients with concomitant left-sided VD are reported in Supplementary Table 4: at univariate analysis, AF, LVEF and PASP were associated with severe TR, while at multivariable analysis only AF (OR 3.08, 95 % CI 1.38–7.04, p = 0.006) remained significantly associated.

Stratified by groups, patient with isolated TR, compared to the other groups, were more often female (66 %), with more history of AF (60 %) and better clinical presentation (NYHA class I in 48 %). Moreover, they presented smaller LV and LA dimensions (mean 45 ± 6 mm and 53 ± 14 ml/m^2^, respectively), higher LVEF (mean 54 ± 10 %), lower PASP (mean 51 ± 16 mmHg) but larger RA dimensions (mean 29 ± 8 cm^2^).

Patients with severe MR + TR were predominantly male (60 %). Compared to the other groups, they had more often history of CAD (34 %) and anemia (81 %) and worst clinical presentation (NYHA class III/IV in 41 %). They were characterized by the largest LV, RV and LA dimensions (57 ± 7 mm, 44 ± 9 mm and 64 ± 15 ml/m^2^, respectively) and by the highest prevalence of severe TR (339, 73 %).

In contrast to the other groups, patients with severe AS + TR presented with older age (mean age 81 ± 9 year), the highest rate of comorbidities (mean CCI 4.7 ± 1.6), worst renal function (mean eGFR 46 ± 20 ml/min/m^2^) and the largest prevalence of severe LV systolic dysfunction (LVEF <40 % in 34 %).

Eventually, severe AR + TR patients, as opposed to other groups, were younger (mean age 73 ± 9 year), more often male (64 %), with lower NYHA class III/IV patients (18 %) and lower rate of comorbidities (mean CCI 3.6 ± 1.5); moreover, they presented both higher SVi (mean 52 ± 19 ml/m^2^) and PASP (mean 58 ± 17 mmHg) values.

The clinical impact of left-sided VD compared to isolated TR patients is shown in Table 2. While the four groups presented with remarkable different clinical impairment, after adjustment for age, gender, CCI and AF, patients with severe MR + TR remained associated with the presence of reduced LVEF, and patients with severe AS + TR with both NYHA class III/IV at presentation and reduced LVEF. Adjustment in a right-side model for PASP, TAPSE and RA pressure showed that both severe MR + TR and severe AS + TR groups remained consistently associated with reduced LVEF, while only severe AS + TR patients with NYHA class III/IV and only patients with severe MR + TR with reduced SVi.

**Table 2:** Clinical consequences of left-sided valvular diseases in the context of tricuspid regurgitation.

TR consequences		Univariate analysis		Adjusted for age, gender, Charlson index, AF		Adjusted for PASP, TAPSE RAP	
Observed	VD group	RR (95 % CI) of consequence	P-value	RR (95 % CI) of consequence	P-value	RR (95 % CI) of consequence	P-value
NYHA class III/IV	Isolated TR	Reference		Reference		Reference	
	Severe MR + TR	1.95 (1.15–3.33)	**0.013**	1.26 (0.70–2.27)	0.422	1.20 (0.62–2.33)	0.573
	Severe AS + TR	3.86 (2.18–6.84)	**<0.001**	3.54 (2.14–5.85)	**<0.001**	2.75 (1.61–4.61)	**<0.001**
	Severe AR + TR	0.95 (0.19–3.25)	0.850				
EF < 50 %	Isolated TR	Reference		Reference		Reference	
	Severe MR + TR	2.47 (1.78–3.43)	**<0.001**	2.20 (1.51–3.20)	**<0.001**	3.96 (2.68–5.85)	**<0.001**
	Severe AS + TR	3.22 (2.07–5.00)	**<0.001**	3.31 (1.99–5.56)	**<0.001**	2.96 (1.54–4.22)	**<0.001**
	Severe AR + TR	2.83 (1.16–6.84)	**0.022**	1.99 (0.67–5.89)	0.210	2.79 (0.84–8.22)	0.064
SVi ≤35ml/m2	Isolated TR	Reference		Reference		Reference	
	Severe MR + TR	1.35 (1.03–1.77)	**0.031**	1.13 (0.77–1.67)	0.502	1.58 (1.06–2.39)	**0.028**
	Severe AS + TR	1.51 (1.08–2.09)	**0.013**	1.93 (0.47–2.69)	0.350	1.42 (0.85–2.39)	0.188
	Severe AR + TR	0.87 (0.55–1.34)	0.540				
GFR <60 ml/min/m^2^	Isolated TR	Reference		Reference		Reference	
	Severe MR + TR	1.27 (0.94–1.71)	0.110				
	Severe AS + TR	1.29 (0.83–2.00)	0.258				
	Severe AR + TR	1.08 (0.70–1.64)	0.403				

Abbreviation as shown in Table 1.

Overall, the survival at 1, 2 and 3 year was 74 % (70–76 %), 65 % (61–68 %) and 57 % (54–61 %) respectively. Three-year survival was significantly different between groups: 62 % (57–68 %), 57 % (53–64 %), 32 % (21–45 %) and 55 % (35–84 %) for isolated TR, for severe MR + TR, for severe AS + TR and for severe AR + TR, respectively.

**Fig. 2:**
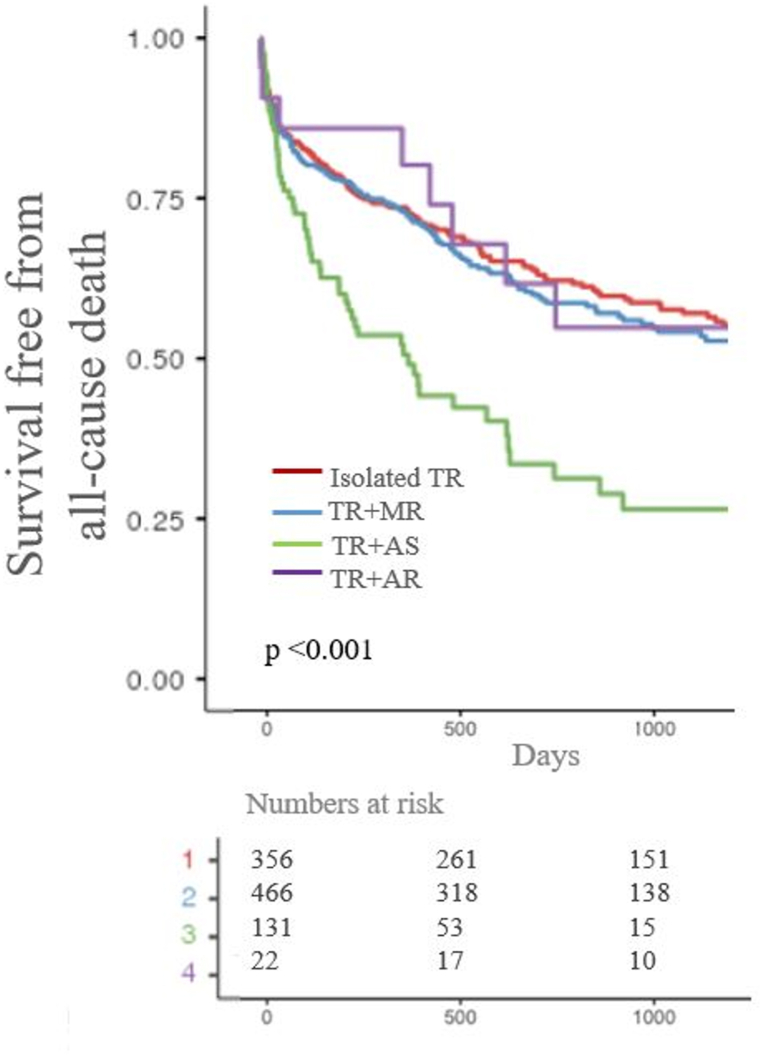
Survival stratified by groups, with follow-up censored at the time of any valvular intervention.

After a median follow-up of 2.7 years, the primary endpoint occurred in 374 (39 %) patients and was significantly more common in patients with severe AS + TR, compared to other groups (p-value <0.001 Fig. 2).

The excessive mortality for severe AS + TR patients compared to isolated TR patients was confirmed at univariate analysis, but not in a model adjusted on age, CCI, LVEF and PASP (see Table 3).

**Table 3:** Univariate and multivariable hazard ratio for mortality.

		All-cause death HR (95 % CI)	p-value
**Univariate**	Isolated TR	Reference	
	Severe MR + TR	1.84 (0.53–2.82)	0.231
	Severe AS + TR	2.39 (1.65–4.72)	**<0.001**
	Severe AR + TR	1.61 (0.77–1.71)	0.471
**Multivariate analysis**	Isolated TR	Reference	
	Severe MR + TR	NA	
	Severe AS + TR	1.98 (0.72–3.55)	0.313
	Severe AR + TR	NA	

Abbreviations as shown in Table 1. Multivariate model adjusted on Age, Charlson Index, PASP, LVEF,.

**Fig. 3:**
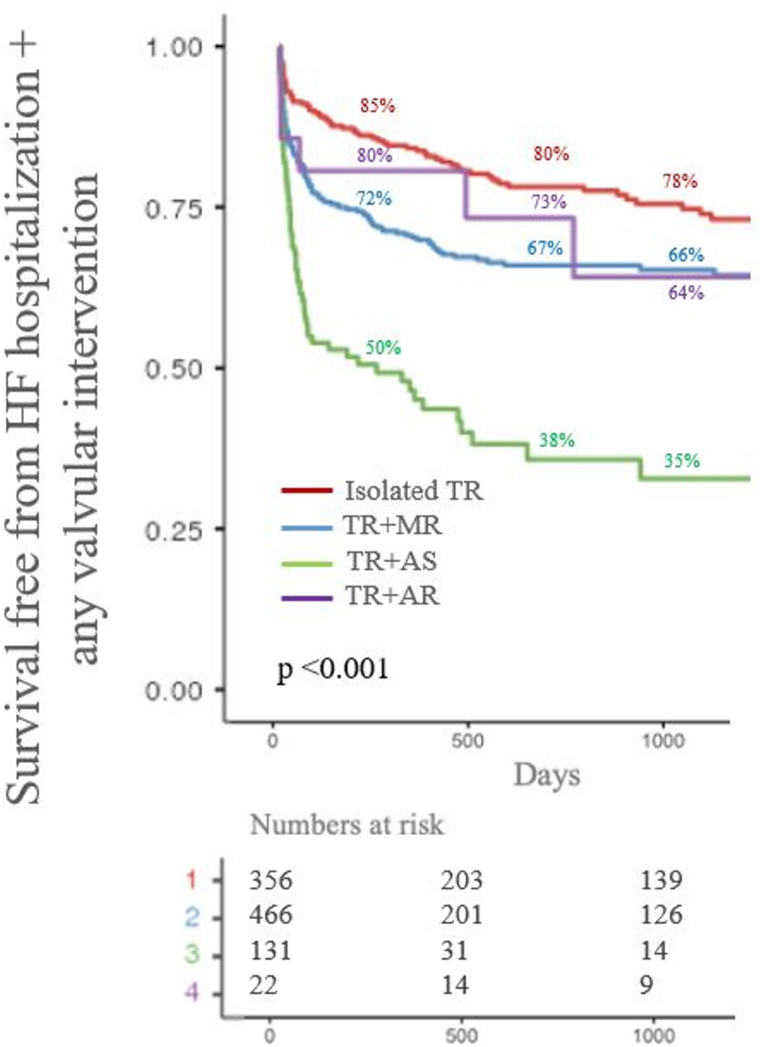
Survival free from the secondary composite endpoint stratified by valvular disease. For each group, percentage of one, two and three-year freedom from the composite endpoint is reported.

During follow-up, overall survival free from the secondary composite endpoint was 74 % (71–77 %), 68 % (65–71 %) and 66 % (63–70 %), at one, two year and three-year respectively. The secondary endpoint occurred in 284 (29 %) patients, significantly more often for patients with severe AS + TR, as reported in Fig. 3 along with one, two and three-year percentages of survival-free from the endpoint for each group.

The predictive value of severe TR on clinical outcomes is shown in Supplementary Table 5: while no significant risk for the primary endpoint was found, severe TR significantly increased the hazard risk for the secondary endpoint at univariate analysis but not in a model adjusted on age, NYHA class, CCI, LVEF and PASP.

Surgical procedures are reported in detail in Table 4. In particular, 173 (18 %) patients underwent valvular intervention during follow-up. Among these, 20 (11 %) underwent concomitant surgical repair (all TV annuloplasty) or replacement of the TV, and 2 (1 %) staged surgical TV intervention. No significant differences between-groups were found in term of TV surgical intervention rate, while only patients with isolated TR were treated with percutaneous TV repair (p overall = 0.030).

**Table 4:** Interventional management of the study population.

	Isolated ≥ moderate TR (n = 356)	TR + severe MR (n = 466)	TR + severe AS (n = 131)	TR + severe AR (n = 22)	p-Value overall
Tricuspid valve management					
Concomitant TV repair or replacement + left side valve intervention (isolated for group 1)	16 (5 %)	16 (4 %)	2 (1.5 %)	0 (0 %)	0.444
Staged TV repair or replacement	0 (0 %)	2 (0.5 %)	0 (0 %)	0 (0 %)	0.624
Transcatheter tricuspid valve repair	6 (2 %)	0 (0 %)^a^	0 (0 %)	0 (0 %)	**0.030**
**Left side valve management**					**p-value** **overall**
Surgical left side valve intervention only, repair or replacement	0 (0 %)	48 (11 %)	9 (7 %)	3 (13 %)	**<0.001**
Transcatheter left side valve intervention only^d^	0 (0 %)	26 (6 %)^b^	48 (37 %)	0 (0 %)^c^	**<0.001**

See previous tables for abbreviations.

a = Indicates p < 0.05 for isolated TR vs severe MR + TR.

b = Indicates p < 0.001 for severe AS vs severe MR + TR.

c Indicates p < 0.001 for severe AS vs severe AR + TR.

d TAVI or edge-to-edge repair.

Patients who underwent valvular intervention, compared to those managed with medical treatment, were younger and with lower mean CCI (both p < 0.001), with better renal function (p = 0.006) and higher mean SVi (p = 0.023), and, although not statistically significant, with higher LVEF and lower PASP (see Supplementary Table 6).

## 4 Discussion

This retrospective multicenter study describes the clinical and echocardiographic features, the outcome and the management of patients with MVD in the context of severe left-sided VD with the additive burden of significant TR.

The main results of the present study are the following:

- Each group of patients with functional TR in the presence of left-sided severe VD presents distinct clinical and echocardiographic profiles, dissimilar from those of isolated TR patients as well.
- Patients with severe AS + TR have considerable worse prognosis; however, after comprehensive adjustment differences in the primary outcome became insignificant, underscoring the serious outcomes of all severe TR.
- Patients with severe left-sided VD and at least moderate TR are profoundly undertreated.

Typically, patients with functional TR due to left-sided VD have been described and managed as a homogeneous cohort. However, the present study demonstrates differences in the prevalence, clinical presentation, outcomes and management.

Patients with isolated TR showed the typical features of this subtype of TR [2,18]: high prevalence of female gender and of AF history, better clinical presentation and larger RA dimensions, lower mean PASP and higher LVEF compared other groups.

Patients with severe MR + TR were the most prevalent in our study. This group showed very specific features: it presented both the worst clinical presentation in term of NYHA class and the largest biventricular and LA dimensions as well as the highest prevalence of concomitant severe TR. Furthermore, this group was associated with an advanced NYHA class at presentation and, even after adjustment for right-side variables, with low Svi and with low LVEF in both adjustment models. Interestingly, while MR conceivably was the leading cause of TR in this group, the development of TR remains an independent predictor of mortality in severe MR patients, whose risk increasing along with the increasing severity of TR [4,5]. Therefore, TR shifts from a marker of advanced disease to a therapeutical target, creating a chicken and the egg-like situation in the late stage of the natural history of this combined VD.

Patients with severe AS and TR were the oldest, with the largest burden of comorbidities, worst renal function and greater % of severely reduced LVEF. In patients with severe AS, the long-term prevalence of at least moderate TR is around 25 % [7]. Although the natural evolution of AS-related cardiac injury still needs to be fully clarified, as recent data suggest that both genetic predisposition and individual susceptibility may play an important role [19]. The involvement of TV apparatus and of right-heart chambers in the extent of AS-related cardiac damage carries important prognostic implications for the outcome of these patients [7,20]. Moreover, ≥ moderate TR has been independently associated with increased risk of all-cause mortality following aortic valve replacement in low-flow low-gradient AS^21^. The marked excess mortality related with TR in the context of severe AS is mirrored in the worst clinical presentation and in the clinical consequences of this combined VD, being it associated with advanced NYHA class and reduced EF in both our adjustment models. These results again underscore the importance of early detecting and of appropriate managing of TR associated with AS, focusing on both the hemodynamic consequences of each concomitant valvular lesion and the clinical context and comorbidities.

The association between AR and TR was the least common, representing 2 % of the whole cohort. There is lack of literature focusing on the epidemiological, clinical and prognostic characteristics of this multiple VD. In the recently published substudy of the EURObservational VHDII Survey [9], the association between severe AR and severe TR was present in 9 patients only (1.8 %) among the cohort of severe left sided VD + severe TR patients. This prevalence is similar to our study, although in this group moderate TR was significantly more common than severe TR. Despite being underrepresented, this group of patients clearly showed favorable characteristics compared to others such as younger age, lower CCI and advanced NYHA class; however, interestingly, patients in this group had the highest mean PASP values, possibly reflecting the chronic long-standing volume and pressure overload typical of this VD [21], leading to PH and to hemodynamically relevant TR.

MVD is usually associated with older age, greater cardiac damage and more comorbidities than single VD [8,9,22]; moreover, due to the high surgical risk of this population, these patients are usually surgically undertreated [7]. The risk of excess long-term mortality of this population is conceivable. Recent insights [10] from the Valvular Heart Disease Survey II, focusing on the outcome of TR in patients with left-sided VD, reported that moderate/severe TR was associated with a worse clinical presentation and poorer outcome at 6 months compared to no/mild TR. In our study, mean age of the entire cohort was 77 ± 9 years, one-third in NYHA class III/IV, mean CCI was 4.3 ± 1.7, 63 % had CKD and 73 % anemia. This complex clinical scenario at presentation largely explains the prevalence of both the primary and the secondary end points during follow-up. Among the four groups, patients with severe AS + TR had the worse prognosis, despite the largest prevalence (44 %) of left-valve interventional treatment; reduced survival compared to other groups was evident since the very first months of follow-up and sustained throughout the years. Of note, excessive mortality for this group, compared to isolated TR patients, was not confirmed in a model adjusted for age, CCI, LVEF and PASP, underscoring the influence of worse clinical/physiologic context in MVD patients and the poor outcomes affecting all types of patients with significant TR [23,24].

Data on the surgical or transcatheter management of patients with multiple VD in the context of significant TR is scarce.

In our multiple VD cohort, whose management represents real-life routine clinical practice, valvular intervention was profoundly underperformed. Namely, only 18 % of the whole cohort was treated with surgical or transcatheter treatment, and only 5 % with TV intervention. In line with recent studies [9,22], our cohort presented a considerable prevalence of cardiac damages and comorbidities, limiting the approach by an invasive treatment; this concept is stressed in Supplementary Table 4, as age, CCI, eGFR and SVi were significantly different between patients who underwent valvular intervention compared to those who did not.

Transcatheter valves interventions have transformed the outcome of patients with VD, particularly in case of high-surgical risk and multiple VD [25]. It must be underlined that transcatheter therapies, especially for atrioventricular valves, were significantly less available during the years when our patients were managed, as they could have increased the percentage of those invasively treated [26].

Our study presents some limitations. First, it is a retrospective study, and suffers from intrinsic pitfalls of this study design. Second, we included patients with moderate TR. However, its independent impact on survival has been now clearly demonstrated [[3], [4], [5]]: therefore, we aimed to investigate the full spectrum of prognostic-relevant TR in the context of concomitant left-sided valve diseases.

The rate of interventional treatments differs according to the specific center as well as the different therapeutical approach. Hence, 73 % of the valvular interventions were performed in one of the two centers: this reflects the real-world nature of this study and could have influenced the number of invasive procedures performed in our cohort. Moreover, we lack information whether the TR has improved with medical treatment over time, another possible explanation of the severe undertreatment of TR in all groups, and regarding echocardiographic follow-up after valvular intervention, as residual valvular disease may have influenced the reported outcomes.

Finally, as most of the echocardiographic studies were performed before 2018, we did not further stratify severe TR patients into massive and torrential grades [27].

## 5 Conclusion

In the context of severe left-sided VD and concomitant significant TR, patients present specific clinical and echocardiographic profiles according to the type of left VD, significantly dissimilar from isolated TR patients as well. Patients with severe AS and ≥ moderate TR experience worse prognosis compared the other groups, but not after comprehensive adjustment for the clinical context. In the overall cohort, valvular surgery was profoundly underused. These results warrant a comprehensive evaluation of these patients at the time of MVD diagnosis to ensure the most appropriate management, particularly in light of current transcatheter treatments.

## Data statement

The data that support the findings of this study are available from the corresponding author, upon reasonable request.

## CRediT authorship contribution statement

**Davide Margonato:** Writing – original draft, Methodology, Formal analysis, Data curation, Conceptualization. **Giacomo Ingallina:** Methodology, Formal analysis, Conceptualization. **Martina Belli:** Investigation, Data curation. **Annamaria Tavernese:** Investigation, Data curation. **Gianluca Ricchetti:** Investigation, Data curation. **Francesco Ancona:** Visualization, Validation. **Stefano Stella:** Visualization, Validation. **Federico Biondi:** Visualization, Validation. **Giorgio Fiore:** Visualization, Validation. **Michele De Bonis:** Validation, Supervision. **Francesco Maisano:** Validation, Supervision. **Yan Topilsky:** Validation, Supervision, Conceptualization. **Eustachio Agricola:** Writing – review & editing, Validation, Methodology, Conceptualization.

## Declaration of competing interest

The authors declare that they have no known competing financial interests or personal relationships that could have appeared to influence the work reported in this paper.
