# Supplementary material for: Tricuspid regurgitation in the context of severe left-sided valvular disease: Patients characteristics and outcome
Source: Heliyon. 2024 Jul 19;10(14):e34874. doi: 10.1016/j.heliyon.2024.e34874 (PMC11325386; doi:10.1016/j.heliyon.2024.e34874)
Supplement: Multimedia component 1 [file mmc1.pdf]

Supplementary Table 1. Baseline characteristics of the original population.

	**Original population (n=1183)**
**Age (years)**	73± 13
**Female gender, n (%)**	581 (49%)
**Weight (kg)**	70± 15
**BMI (kg/m²)**	25.6± 4.9
**BSA (m²)**	1.8± 0.2
**Hypertension**	792 (67%)
**Diabetes**	272 (23%)
**Dyslipidemia**	495 (42%)
**Smoking history (former or current)**	136 (11%)
**History of CAD**	318 (27%)
**Previous CABG**	40 (3%)
**Cardiomyopathy**	65 (5%)
**CIED**	151 (13%)
**Atrial fibrillation**	533 (45%)
**COPD**	88 (7%)
**History of cancer (past or active)**	177 (15%)
**CKD**	734 (62%)
**Charlson Comorbidity Index**	4.2± 2.2
**NYHA class**	
**I**	343 (29%)
**II**	438 (37%)
**III/IV**	402 (34%)
**Hb (g/dl)**	11.1± 2
**eGFR (CKD-EPI, ml/min/mq)**	53± 27
**LVEF (%)**	52± 13
**SVi (ml/m2)**	33± 11
**PASP (mmHg)**	50± 16
**RVEDD (mm)**	42± 8
**TAPSE (mm)**	18± 5
**VC (mm)**	7± 3
**RAES area (cm2)**	26± 8
**RAP (mmhg)**	10± 6

Values are mean ± SD or n (%), unless otherwise specified. Abbreviations: BMI, Body Mass Index, is the weight in kilograms divided by the square of the height in meters; BSA, Body Surface Area; CAD, Coronary Artery Disease; CABG, Coronary Artery Bypass Graft; CIED, Cardiac Implantable Electronic Device; COPD, Chronic Obstructive Pulmonary Disease; CKD, Chronic Kidney Disease, defined as eGFR <60 mL/min; eGFR, estimated Glomerular Filtration Rate; EPI, Epidemiology Collaboration; LVEF, Left Ventricular Ejection Fraction; SVi, Stroke Volume Indexed; PASP, Pulmonary Artery Systolic Pressure; RVEDD, Right Ventricle End-Diastolic Diameter; TAPSE, Tricuspid Annular Plane Systolic Excursion; VC, Vena Contracta; RAES, Right Atrium End-Systolic; RAP, Right Atrium Pressure.
