# Supplementary material for: Tricuspid regurgitation in the context of severe left-sided valvular disease: Patients characteristics and outcome
Source: Heliyon. 2024 Jul 19;10(14):e34874. doi: 10.1016/j.heliyon.2024.e34874 (PMC11325386; doi:10.1016/j.heliyon.2024.e34874)
Supplement: Multimedia component 2 [file mmc2.pdf]

Supplementary Table 2. Propensity score matching for age was used to create matched cohorts in the different groups of VD with an absolute standardized differences <10%

	Overall population	Isolated TR	TR + severe MR	TR + severe AS	TR+ severe AR	Absolute standardized difference
**Number of patients**	1183	423 (36%)	599 (51%)	132 (11%)	29 (2%)	
**Mean Age (years)**		82	67	62	74	25%
**Propensity age exact**						
**Number of patients**	975	356 (37%)	466 (48%)	131 (13%)	22 (2%)	
**Mean Age (years)**		81	73	76	78	9%
