# Supplementary material for: Tricuspid regurgitation in the context of severe left-sided valvular disease: Patients characteristics and outcome
Source: Heliyon. 2024 Jul 19;10(14):e34874. doi: 10.1016/j.heliyon.2024.e34874 (PMC11325386; doi:10.1016/j.heliyon.2024.e34874)
Supplement: Multimedia component 3 [file mmc3.pdf]

Supplementary Table 3. Differences in TR severity prevalence in the overall population and of patients according to valvular heart disease

	**Overall population (n=975)**	**Isolated TR (n= 356, 37%)**	**Severe MR+TR (n= 466, 48%)**	**Severe AS+TR (n=131, 13%)**	**Severe AR+TR (n= 22, 2%)**	**p-value for Pairwise Comparison Analysis**					
**Moderate TR**	502 (52%)	250 (70%)	127 (27%)	105 (80%)	20 (91%)	Isolated TR vs Severe MR+TR	Isolated TR vs Severe AS+TR	Isolated TR vs Severe AR+TR	Severe MR+TR vs Severe AS+TR	Severe MR+TR vs Severe AR+TR	Severe AS+TR vs Severe AR+TR
**Severe TR**	473 (48%)	106 (30%)	339 (73%)	26 (20%)	2 (9%)	**<0.001**	**0.038**	0.066	**<0.001**	**<0.001**	0.363
