# Supplementary material for: Tricuspid regurgitation in the context of severe left-sided valvular disease: Patients characteristics and outcome
Source: Heliyon. 2024 Jul 19;10(14):e34874. doi: 10.1016/j.heliyon.2024.e34874 (PMC11325386; doi:10.1016/j.heliyon.2024.e34874)
Supplement: Multimedia component 4 [file mmc4.pdf]

Supplementary Table 4. Univariate and multivariate analysis of factors associated with severe TR in patients with concomitant left-sided VD.

**Parameter**	**Univariate**		**Multivariate**	
	**OR (95% CI)**	**p value**	**OR (95% CI)**	**p value**
Age	1.01 (0.97-1.03)	0.159		
Gender (female vs male)	0.74 (0.53-1.04)	0.079		
History of CAD	1.32 (0.89-1.97)	0.180		
AF	3.26 (1.46-7.27)	**0.004**	3.02 (1.38-7.04)	**0.006**
COPD	1.30 (0.80-1.99)	0.232		
LVEF (%)	1.03 (1.01-1.04)	**0.002**	1.01 (0.99-1.03)	0.251
PASP (mmHg)	1.01 (1.00-1.03)	**0.021**	1.01 (0.98-1.04)	0.280
