# Supplementary material for: Tricuspid regurgitation in the context of severe left-sided valvular disease: Patients characteristics and outcome
Source: Heliyon. 2024 Jul 19;10(14):e34874. doi: 10.1016/j.heliyon.2024.e34874 (PMC11325386; doi:10.1016/j.heliyon.2024.e34874)
Supplement: Multimedia component 5 [file mmc5.pdf]

Supplementary Table 5. Univariate and multivariable hazard ratio of severe TR for the outcomes.

		All-cause death		HF hospitalization + valvular intervention	
		**HR (95% CI)**	**p-value**	**HR (95% CI)**	**p-value**
**Univariate**	Severe TR	1.18 (0.82-1.71)	0.364	2.36 (1.27-3.52)	**0.008**
**Multivariate analysis**	Severe TR	NA		1.12 (0.78-1.58)	0.447

Multivariate analysis– Adjusted for Age, NYHA class, Charlson index, LVEF, PASP
