# Supplementary material for: Tricuspid regurgitation in the context of severe left-sided valvular disease: Patients characteristics and outcome
Source: Heliyon. 2024 Jul 19;10(14):e34874. doi: 10.1016/j.heliyon.2024.e34874 (PMC11325386; doi:10.1016/j.heliyon.2024.e34874)
Supplement: Multimedia component 6 [file mmc6.pdf]

Supplementary Table 6. Baseline differences between patients divided according to therapeutical management.

	**Valve intervention (n=176, 18%)**	**Medical treatment (n= 799, 82%)**	**p-value**
Age (years)	74±9	78±9	**<0.001**
Charlson Comorbidity Index	3.7±1.4	4.5±1.7	**<0.001**
eGFR (ml/min/mq)	53±21	47±25	**0.006**
PASP (mmHg)	50±15	52±16	0.762
LVEF (%)	53±14	51±13	0.351
Stroke Volume Indexed (ml/m2)	37±11	33±11	**0.023**

Values are mean ± SD or n (%), unless otherwise specified. See previous tables for abbreviations.
